# Editorial: Inference of Biological Networks

**DOI:** 10.3389/fbinf.2021.821978

**Published:** 2022-01-06

**Authors:** Tatsuya Akutsu, Hongmin Cai

**Affiliations:** ^1^ Bioinformatics Center, Institute for Chemical Research, Kyoto University, Kyoto, Japan; ^2^ School of Computer Science and Engineering, South China University of Technology, Guangzhou, China

**Keywords:** glycosylation networks, metabolic networks, protein-protein interaction networks, biological networks, bioinformatics

In living cells, various kinds of molecules interact with each other. These interactions constitute several types of biological networks such as protein-protein interaction networks, gene regulatory networks, and metabolic networks. In order to study these networks, one needs to identify the key driving structures of these networks from experimental data or literature. To this end, various computational methods have been proposed. However, existing methods are not sufficient, and new technologies such as single cell analysis are becoming widely available. Therefore, it is strongly needed to develop new computational methods for inference of biological networks and/or molecular interactions. Furthermore, how to utilize inferred and/or known networks to enhance understanding of these interactions is also an important issue.

All articles in this topic highlight inference and/or utilization of biological networks. Krishnan and Krishnan studied the glycosylation network related to the spike (S) protein of SARS-CoV-2. They reconstructed the glycosylation network based on previously published mass spectrometry data using an existing glycosylation network analysis tool, GNAT. Using this reconstructed data, they simulated the effect of blocking enzymes, swainsonine or deoxynojirimycin for blocking mannosidase-II and indolizidine for blocking alpha-1,6-fucosyltransferase. As a result, it is suggested that these enzymes play important roles in the glycan biosynthesis pathway, and without them, the glycans synthesized are altered, changing the glycoprotein profile. The results and data might be useful for future studies on heterogeneity in the N-glycan profile of the spike (S) protein of SARS-CoV-2 and its potential effect on vaccine efficacy or adverse reactions to the vaccines.

Ma and Tamura studied metabolic networks. They considered the problem of calculating reaction deletion strategies that achieve growth coupling of designated target metabolite production in a flux-balance model of metabolic networks. They developed the DynCubeProd method, which is an improved version of the CubeProd method. Although CubeProd exhaustively divides the solution space based on pre-specified parameters, DynCubeProd dynamically and gradually divides the solution space into smaller and smaller pieces. The power of DynCubeProd was demonstrated by comparing with CubeProd and other tools using large-scale metabolic networks. In particular, DynCubeProd was more than 10 times faster than CubeProd for some well-known metabolic network model.

Martines et al. studied protein-protein interaction (PPI) networks. In order to infer new PPIs from multiple data sources, they propose the PredPri workflow that enables prediction of PPIs by combining multiple kinds of evidence, including the structure, sequence, and functional annotations, using boosting and stacking machine learning techniques. They also propose the PPIVPro pipeline for validating predicted PPIs, based on cellular co-localization filtering and a focused search of PPI evidence on scientific publication. The proposed methods were shown to be useful by means of comparison with several recent tools.

We believe that all of these works are valuable contributions to the study of biological networks and will stimulate further developments and applications.

